# The Evolution of Patient Empowerment and Its Impact on Health Care’s Future

**DOI:** 10.2196/60562

**Published:** 2025-05-01

**Authors:** Bertalan Mesko, Dave deBronkart, Pranavsingh Dhunnoo, Nora Arvai, Gellért Katonai, Sara Riggare

**Affiliations:** 1 The Medical Futurist Institute Budapest Hungary; 2 e-Patient Dave, LLC Nashua, NH United States; 3 Department of Computing Atlantic Technological University Donegal Ireland; 4 Participatory eHealth and Health Data Research Group, Department of Women’s and Children’s Health Uppsala University Uppsala Sweden; 5 Centre for Disability Research Uppsala University Uppsala Sweden

**Keywords:** patient empowerment, patient design, foresight, future studies, health care future, patient autonomy

## Abstract

In the 21st century, health care has been going through a paradigm shift called digital health. Due to major advances and breakthroughs in information technologies, most recently artificial intelligence, the patriarchy of the doctor-patient relationship has started evolving toward an equal-level partnership with initial signs of patient autonomy. Being an underused resource for centuries, patients have started to contribute to their care with information, data, insights, preferences, and knowledge. It is important to recognize that at its core, digital health represents a cultural transformation, where patient empowerment has likely played the most significant role in driving these changes. This viewpoint paper traces the remarkable journey of patient empowerment from its nascent stages to its current prominence in shaping health care’s future. Spanning over two and a half decades, we explore pivotal moments and technological advancements that have revolutionized the patient’s role in health care. We dive into a few historical milestones, mainly in the United States, that have challenged and redefined societal norms around agency, drawing parallels between patient empowerment and broader social movements, such as the women’s suffrage and civil rights movements. Through these lenses, we argue that patient empowerment is not solely a function of knowledge or technology but requires a fundamental shift in societal attitudes, policies, health care culture, and practices. As we look to the future, we posit that the continued empowerment of patients will play a pivotal role in the development of more equitable, effective, and personalized health care systems. This paper calls for an ongoing commitment to fostering environments that support patient agency, access to resources, and the realization of patient potential in navigating and contributing to their health outcomes with an emphasis on the emerging significance of patient design.

## Introduction

Since Hippocrates, the expectation has been that patients lack the necessary knowledge and are not capable of taking part in their own health or disease management. This is very different to what we see today, when patients express informed preferences about treatment options, or know of important journal papers their physicians might have not seen. With the rise of social media and the appearance of digital health technologies, the ivory tower of medicine has started to break down, and new kinds of partnerships have started to form between medical professionals and their increasingly empowered patients [1].

In the 21st century, health care has been going through a paradigm shift called digital health [2]. Being an underused resource for centuries, patients have started to contribute to their care with information, data, insights, preferences, and knowledge. Due to major advances and breakthroughs in information technologies, the patriarchy of the doctor-patient relationship has started evolving toward an equal-level partnership with initial signs of patient autonomy.

Examining trends that might have contributed to this phenomenon, we observe several key developments: wearable sensors have become accessible to hundreds of millions of patients; digital patient communities have connected individuals globally; and, more recently, generative artificial intelligence (AI) has started to become a widely accessible tool for patients [3].

These observations suggest that a technological revolution has been impacting health care. However, it is important to recognize that at its core, digital health represents a cultural transformation, where patient empowerment has likely played the most significant role in driving these changes. To understand the remarkable progress of empowerment, and the forces that drive it, we must first understand the functional meaning of the word.

In 2002, the World Bank [4] defined empowerment as increasing people’s capacity to make choices and convert them into actions and outcomes. A 2023 brief significantly expanded this, articulating the need for resources and the need for social change such that people want to take action, are equipped, and are socially allowed to [5]. This was described in three pillars: (1) resources: providing people the tools, the assets, and the information to be able to follow through with a self-governed goal; (2) agency: the person’s ability to think critically, and willingness to take their lives into their own hands, “indicative of their level of self-efficacy and self-esteem”; and (3) context: a cultural environment that allows expressing and pursuing desires, including an equitable decision-making environment.

In this viewpoint paper, we will give an overview of the past 25 years of patient empowerment based on the World Bank’s 3 pillars. We will also outline what it means for health care’s near future and offer our vision about how it might unfold in the next 25 years.

## The Evolution of Patient Empowerment: A Brief Historical Perspective

### Overview

The journey of patient empowerment relates to several factors: the evolution of knowledge dissemination, shifts in the doctor-patient relationship, the acknowledgment of agency, social change, and technological advancements. This narrative not only reflects progress in health care but is also an expression of a broader societal transformation toward equity, autonomy, and participation.

“Knowledge itself is power,” said Francis Bacon, and indeed, empowerment’s story has followed the spread of knowledge. The Garden of Eden cautioned against knowledge but with the revolutionary invention of Gutenberg’s press in 1450, mass dissemination of knowledge began. The spread of literacy signified a pivotal shift in authority from the gatekeepers to the individual—a sequence that would play out centuries later when medical knowledge escaped its gatekeepers and became available to individuals via the web. It was the single most transformational moment in the history of patient power.

### Resources: How Technological Progress Has Fueled Patient Empowerment

The rise of patient empowerment has been facilitated by a number of technological advancements that marked the digital health age [6]. The dissemination of the internet during the 20th century can be considered as the first major technological progress that would eventually lead to today’s empowered patients. The internet democratized access to information, for example, health-related information [7], which had traditionally been restricted to the ivory towers of health care professionals.

Apple’s launch of the first iPhone in 2007 ushered in the dawn of modern smartphones. Their subsequent adoption enabled patients to access health information and digital patient communities in a more accessible fashion, from anywhere with an internet connection. Soon after, smartphone app stores would launch and offer dedicated apps tailored for condition management. These provided further patient autonomy by enabling patients to record and collect their personal health data, which they could present during medical visits.

Then, the rise of digital health in the 2010s enabled by tools like digital thermometers, blood pressure cuffs, and mobile health apps revolutionized patient self-monitoring and situational awareness, thereby empowering patients to take an active role in their health management [8]. Patients in communities could compare experiences in an evidence-informed way, never before possible. This new type of knowledge created a new type of power.

One of the major technological advancements to make an impact in the late 2010s and early 2020s is AI algorithms, even before generative AI arrived with large language models such as ChatGPT [3]. Such smart software was integrated into hardware such as the AliveCor personal electrocardiogram sensors as well as into symptom checkers to automate processes and provide individual insights.

The digital health landscape and patient empowerment are bound to be further impacted by new technological developments in the upcoming years and decades. Already, generative AI tools are showing promise in the health care setting, and patients will likely benefit from such advanced technologies whether it is to inform their health care management or share insights with the broader medical community.

### Agency: Knowledge Is Power

The transformative impact of all these technological changes was that for the first time in history, patients could have self-knowledge beyond what their physicians have. They still needed to learn what to do with it, in precisely the same way that Gutenberg’s books needed literate readers. However, knowledge is power, and doors opened to a new future.

Sociologically, the essence of empowerment lies in the concept of agency—the capacity to act independently and make one’s own choices. This principle is crucial in health care, where access to information, including medical records, patients’ health data, and the medical literature, is fundamental for both patients and doctors to reach their potential. The struggle for agency among suppressed classes in broader society, such as women and minorities, parallels the fight for patient empowerment, illustrating a broader societal challenge against paternalistic and exclusionary attitudes.

No matter how remarkable the progress we have witnessed in health care technology, the adoption rate of new technologies within the traditional health care setting always lagged behind other industries [9]. This meant a slower rate of adopting novel approaches that would be beneficial, even lifesaving, for patients. This slow penetration of useful new tools forced patients to take matters into their own hands, with the assistance of communication channels that the technological revolution enabled.

This is exemplified by the OpenAPS (the open-source automated pancreas system) project, initiated by the digital diabetes community in response to the slow-paced nature of regulatory bodies, clinical trials, and commercialization efforts [10]. Its aim was to make a safe, effective do-it-yourself artificial pancreas system accessible to type 1 diabetes worldwide. This system automatically adjusts insulin pump delivery to maintain safe blood glucose levels overnight and between meals. Dissatisfied with existing glucose monitoring systems, patients took matters into their own hands, leveraging open-source software and data from their on-body devices to create a solution that surpassed existing industry standards. The first do-it-yourself closed loop device that inspired OpenAPS was built in 2014, at a time when commercial alternatives were still years away from being available [11]. Initiated by Lewis et al [12] due to dissatisfaction with existing glucose monitoring systems, this project was the genesis of the #WeAreNotWaiting hashtag, showcasing patients’ capabilities to drive innovation and develop solutions that surpass the existing scientific and industry standards. This initiative underscores the power of technological advancements, patient agency, and information sharing, with patients driving innovation for a better and safer life.

### Context: The Long-Term Impact of Patient Empowerment on the Doctor-Patient Relationship

Meanwhile, in parallel to the technological changes, cultural initiatives were introduced, such as the Patients Included movement represented by the #PatientsIncluded social media hashtag, which directly confronted the Hippocratic paradigm. By adhering to Patients Included [13] charters, organizations can display their commitment to integrating patient input into their activities. This approach has since been adopted by medical advisory boards, academic journals, and conferences, indicating the recognition of patients’ valuable insights in health care decision-making as well as product innovation and even government policy-making. Such a change in dynamic, in part thanks to novel technologies that democratize access to knowledge, communication, and concurrent social change, is a hallmark of the digital health age.

The dynamics of the doctor-patient relationship have also seen profound changes, evolving from ancient healers’ exclusive access to spiritual insights about diseases to a more scientific yet paternalistic model up until the late 1800s. The depiction of this relationship in the 1891 painting “The Doctor” illustrates a compassionate yet helpless figure, unable to save a child due to the limited scientific knowledge of the time. This relationship underwent scrutiny and philosophical debates, notably by Szasz and Hollander [14], and further by Emanuel and Emanuel [15], who explored models ranging from paternalistic to partnership-based approaches, marking a gradual shift toward recognizing the patient as an active participant in their health care.

Since the doctor-patient relationship is a system, a change in one of its members will affect the other as well. The empowered patient has resulted in new medical roles, such as mentor, guide, or coach. As the role of patients changed during the paradigm shift, so did the medical identity. This change holds the promise of more effective health care delivery, resulting in more successful treatment of patients, greater patient satisfaction, and a more fulfilling medical profession.

While such a paradigm shift in health care was enabled by technological means, it really represents a cultural transformation in how health care stakeholders participate in medical decisions: no amount of knowledge or data will transform anything if it is culturally forbidden to enter the conversation. This is the third pillar of the World Bank’s framework: a context of cultural permission. By accepting patients as equal-level partners in the medical team, the evolving medical culture opens the door for them to meaningfully contribute to health care matters. It empowers them.

This means that in contemporary medical practice, health care professionals face new responsibilities and challenges. As the cultural evolution continues to unfold, and considering the unique informational needs of each patient, physicians must evaluate and determine, during the initial encounter, how to communicate effectively with the individual before them. This includes assessing the extent to which the patient should be involved in decision-making as well as the level of detail required when explaining potential treatment options and outcomes. Such considerations are pivotal, as the establishment of mutual understanding essentially shapes the patient’s satisfaction, sense of importance, and perception of shared collaboration toward a common therapeutic goal.

More recently, public and patient involvement (PPI) has become an increasingly recommended practice in health care research [16]. Through PPI input, patients become further involved in academic activities as part of the research team. They can assist in the research design, analyzing data and improving recruitment rates. Such involvement has been successfully integrated across various research levels, from doctoral research programs to feasibility assessments of national programs [17]. The latter is exemplified by the Improving Diabetes Eye Screening Attendance study [18]. It included PPI input throughout the research duration to test the feasibility of an intervention aimed at improving the uptake of the national diabetic retinopathy screening program in Ireland. This is in stark contrast to traditional research where patients were not as involved, save for the collection of data. Through approaches such as PPI, patient involvement as active members of research teams is being valued for their meaningful contribution.

The rising importance of patient involvement in research has been further explicitly supported and incentivized by policy makers worldwide [19]. The Australian National Health and Medical Research Council has, since 2005, established guidelines for the involvement of consumer and community in health research. Similarly, the United Kingdom’s National Institutes of Health Research has issued guidance to support public involvement in health and social care research [20]. In the United States, the government-sponsored Patient-Centered Outcomes Research Institute was set up in 2010 to fund patient-centered comparative clinical effectiveness research [21]. Such research can provide deeper insights into the patients’ worldview with the aim to improve the research process itself. The attention and support provided by government bodies for patient-centered research and patient involvement indicate that patients’ input is being increasingly valued. This further highlights the cultural transformation brought on by the transition to the digital health era.

Guidance and support from policy makers have further encouraged individual health care institutions to integrate patient input into their organizational efforts. For example, the German Center for Diabetes Research includes in its organization a Citizens’ and Patients’ Advisory Board [22]. The members advise the research center, from the perspective of citizens and patients, on aspects ranging from translational research strategy to research projects.

In Denmark, the Danish Cancer Society has stood out as being one of the first patient organizations to make specific requirements for PPI in applications for research grants [23]. The range of patient input, from determining research grant awardees to the quality of care in hospitals, indicates the growing responsibilities that patients are trusted with. As we progress further into the digital health era, we can expect similar levels of high-level responsibilities across more health care organizations.

Over time, we have transitioned away from viewing the healer, known as the “shaman” in ancient times, as someone with a connection to higher powers and the ability to communicate with spirits. This relationship served as the perfect base for traditional paternal medicine. Now, it is gradually being replaced by a more human-centered, egalitarian, and mutual relationship, where patients also play an active role in shaping their fate and health.

## Discussion and Future Directions

### Overview

This historical perspective underscores the remarkable journey of patient empowerment, from restricted knowledge and paternalistic health care relationships to a new era of autonomy, agency, and innovation driven by patients themselves. The story of patient empowerment is a beacon of progress, illustrating the power of knowledge and real-time data in the broader context of societal evolution toward inclusivity, participation, and respect for individual agency.

We note that patient empowerment has evolved in accordance with the World Bank’s pillars:

- Resources: Patients cannot be empowered without information; today, through the internet, digital health, and most recently, generative AI, empowerment become possible. But information alone is not enough.
- Agency: Patients cannot be empowered if they do not want to speak up or do not know how; now, our culture’s decades of social change have produced people who are willing to speak up if they know that they can (but they will be impotent without information resources).
- Context: Patients will still not be empowered even if they have desire and knowledge unless society welcomes their voices. The progression of global social movements has led to society listening—the “context” according to the World Bank.

In the past 25 years, the explosion of knowledge, data, and digital health tools has had a transformational effect on patients’ capacity to know their situation and learn about it. This is the essence of patient empowerment: increasing these capacities, so that people have the power to pursue what is important to them, and the change has gone beyond that: now, there are even patients who invent treatments.

Paternal practices based on the old paradigm can still suppress the expression of new possibilities. Achieving health care’s new potential in the 21st century will require optimizing for empowered patients, which in turn requires suppressing paternal practices. We must instead build a new paradigm, a new evidence base, by rigorously documenting case after case, where patients in the digital era have violated the old paradigm by contributing real value to their health, with or without physicians’ help.

In summary, the dynamic shift between patients and health care professionals indicates the erosion of the traditional paradigm. It relied on the assumption that patients’ inputs in health care decisions were not as valuable as that of health care professionals. This led to the hierarchical model of the doctor-patient relationship that persisted throughout the history of medicine. Physicians would make the decisions regarding patients’ medical journey, and patients had to follow the prescribed pathway, with little to no input of their own. This model assumed that patients, lacking clinical expertise and medical knowledge, would not bring valuable contributions in devising their health care experience and that such input might even be harmful.

### Does Increased Empowerment Lead to Better Health Outcomes?

Empowered patients exhibit better management of chronic conditions, including diabetes, hypertension, asthma, and endometriosis, through enhanced self-care behaviors and adherence to treatment regimens [24,25]. Empowering patients to actively engage in shared decision-making processes has been linked to more favorable treatment outcomes and higher rates of treatment adherence, thereby underscoring the significance of patient involvement in care planning and management [26,27]. This facilitates the delivery of patient-centered care, where health care decisions are aligned with individual values and priorities. Furthermore, empowered patients are better equipped to navigate complex health care systems, leading to improved care coordination and continuity across care transitions, which is a crucial skill in a system where patients still often feel lost and uncertain.

Enhanced empowerment has also been linked to higher levels of patient satisfaction, as individuals perceive greater control over their health care experiences and outcomes [28,29]. Empowered patients report feeling more informed, supported, and respected by health care providers, leading to increased trust and confidence in the care received. Involvement in decision-making processes empowers patients to voice their preferences and concerns, fostering a sense of agency and partnership in their health care journey.

Health care providers can also benefit from increased patient empowerment, as it promotes adherence to evidence-based practices, facilitates in-time access to preventive services, and supports collaborative care planning efforts [30]. Additionally, empowering patients to take an active role in managing their health promotes the efficient use of health care resources, leading to cost savings and optimized health care delivery workflows. In a health care system where doctors and patients collaborate toward a common goal, and thanks to digital technology, they have a bit more time for this work, where patients are more satisfied and express this joy, and doctors are much less exposed to the dangers of overwhelming and burnout.

Despite its clear benefits, patient empowerment faces numerous challenges and barriers. These include disparities in every area of the World Bank’s framework: access to health care resources, inadequate health literacy (which is part of agency—ability to act), and cultural barriers and resistance from entrenched medical practices (context).

It must be emphasized that just as in the women’s movement, transforming relationship roles will require new ways of thinking by both parties. In medical education, very little attention is paid to empowered patients (e-patients), so doctors are often unprepared to deal with them; this must be changed. Since new doctors are often still socialized in a patriarchal system, patients who dare to ask questions or seek explanations are often perceived as a threat to their own expertise. In addition, fully optimized adoption of digital technologies in patient empowerment will require resolving various ethical, legal, and social concerns. Privacy issues, ethical dilemmas, data security breaches, and the potential to widen health disparities are significant considerations.

By addressing these challenges and advancing our understanding of empowerment within health care, we can harness its potential to optimize patient care and improve health outcomes. In response to the mentioned challenges, various solutions have been already proposed. There are efforts to minimize disparities in digital access, aiming to improve internet infrastructure in underserved areas and promote digital literacy among marginalized populations.

Health literacy interventions encompass digital health literacy programs, whose goal is to equip individuals with the skills to critically evaluate digital health information (and their own health data) to be able to make shared decisions about their health.

To overcome cultural barriers, strategies involve culturally tailored digital health interventions and the integration of cultural competency training into medical education curricula. Some supportive material already exists for doctors to prepare them for collaboration with e-patients, and our paper also aims to raise awareness of the necessity of education in this regard.

Addressing resistance from medical professionals requires systematic changes, including shifting toward patient-centered care models and promoting interdisciplinary collaboration between health care professionals and digital health experts.

### Future Perspectives: From Patient Empowerment Toward Patient Design and Patient-Driven Innovation

Patient empowerment evolving into patient design is the hallmark of how health care systems are engaging more meaningfully with the patient perspective [31]. Empowerment, as the initial step, enabled patients to take an informed, active role in their own care. Building upon this, the patient design approach invites patients to be a collaborative partner, directly involved with decision-making at an executive level.

Over the past decade, “patient centricity” became a central mantra for many health care companies, adopting “patient-first” as a guiding principle. The shift was foreseeable, given that the democratization of health care has empowered patients to expect accessible and reliable information as well as intuitive tools and supportive services. Policy makers have also responded to this growing patient empowerment movement. In 2017, the Food and Drug Administration launched the Patient Engagement Advisory Committee to better integrate the patient perspective into regulatory decision-making. In Europe, Patient Engagement Resource Center was founded in 2020, being a repository of publicly available tools that support patient’s engagement in health care research.

For the cultural shift to be fully manifested, we must strive for an actively collaborative process when it comes to designing health care. In the patient-centric model, patients are merely passive actors in the process, their opinion is heard, but they have little to no executive power in the final decision.

In contrast, patient design means patients are elevated to the highest levels of decision-making, where they can shape and advise the strategic direction from being board members. This “co-design” approach acknowledges that patients are the true experts on their own experiences and needs, and their creative contributions are essential to enhancing the value and quality of health care services.

Patient design is already transforming health care in tangible ways. Studies on patient-driven innovation suggest that this emerging field holds great potential [32,33].

### Recommendations

To facilitate the transition toward patient empowerment, we summarize several recommendations that health organizations, companies, and governments might want to consider:

#### Promote Digital Literacy

Recommend that health care providers and governments offer programs to enhance digital literacy among patients and even among primary school students as part of health education. This ensures that patients can effectively use digital health tools, which are pivotal in patient empowerment.

#### Implement Shared Decision-Making

Advocate for the adoption of shared decision-making protocols within health care settings to ensure that patients are actively involved in their health care decisions, thereby fostering a sense of empowerment. Large language models may be adapted as “mediation” tools, negotiating trade-offs without physician burden.

#### Increase Patient Access to Personal Health and Medical Data

Suggest (or even mandate) that health care providers implement systems that allow patients easy and secure access to their own medical records, as is mandatory today in the United States. Access to personal health data can enhance self-management and decision-making capabilities because knowledge is power and is fuel for AI. Encourage interoperability of health data to further expand the ecosystem of patient-empowering apps.

#### Train Health Care Professionals How to Work With Empowered and Informed Patients

Recommend ongoing training for health care providers in areas such as empathy, communication, and cultural competence to improve interactions with patients, making them more effective and supportive of patient empowerment. Teach both patients and professionals the methods of a knowledgeable, collaborative relationship.

#### Develop Policy for Patient-Centric Care

Recommend that policy makers develop and enforce regulations that support patient-centric care practices, ensuring that health care systems prioritize patient empowerment in both philosophy and practice.

#### Support Patient Design and Patient-Driven Innovation

Encourage health care organizations to integrate patient design in the development of new services, tools, and care pathways to ensure that these are aligned with the actual needs and preferences of patients. Do this by seeking out the new paths being hewn by pioneering patients.

Table 1 summarizes these findings and future directions.

With the ongoing and earlier-mentioned cultural changes, the wide adoption of generative AI, and the rise of digital health, we have all the reasons to expect patient empowerment to further advance the field of medicine and keep on shaping the health care landscape too.

Patient empowerment has been an unprecedented driving force behind health care transformation in the last 25 years, and it is reasonable to be optimistic about its ongoing impact on the doctor-patient relationship especially in an age where technologies advance so fast and providing health care relies more and more on their use.

**Table 1:** Key concepts and impacts of patient empowerment in health care.

Key concepts			Impacts
**Core components**			
	Agency	Ability to make choices and take actions.	
	Resources	Tools, information, and assets.	
	Context	Supportive cultural and social environment.	
**Technological drivers**			
	Internet and smartphones	Access to information and communities.	
	Wearable devices	Enhanced self-monitoring and data collection.	
	Artificial intelligence	Personalized health care insights and language processing.	
	Telemedicine	Access to care anywhere.	
**Impact on health care**			
	Improved self-management	Better chronic condition management through much greater continuous awareness and treatment adherence.	
	Higher patient satisfaction	Increased trust, communication, ability to pursue autonomous goals, and involvement.	
	Enhanced collaboration	Better rapport and patient-provider relationship.	
	Cost savings and efficiency	Optimized resource use.	
	Reduced hospital readmissions	Better outpatient care and follow-up.	
	Improved health literacy	Better informed patients about their conditions and treatments.	
	Increased preventive care	More proactive health measures and screenings.	
	Enhanced patient safety	Fewer medical errors through better-informed patients.	
	Greater accessibility to care	Remote and underserved areas benefit from telehealth.	
	Enhanced patient advocacy	Patients are increasingly able to take an active role in their health decisions.	
	Increased research participation	More patients involved in clinical trials and research both through increased patient design or approval of trials and through a greater ability to match individuals with specific trials.	
**Future directions**			
	Support patient design and patient-driven innovation	Include patients in designing all aspects of health care services.	
	Regulatory reforms	Create laws that support patient innovation and autonomy.	
	Train health care professionals	Train providers in supporting patient agency, especially how to work with an equipped, informed patient.	
	Implementation of shared decision-making	Involve patients in health care decisions through an increasingly collaborative care relationship.	
	Promote digital literacy	Teach digital health tool use to patients, including effective use of artificial intelligence.	

## Abbreviations

AI: artificial intelligence
OpenAPS: open-source automated pancreas system
PPI: public and patient involvement

## References

[1] Steinhubl SR, Muse ED, Topol EJ (2013). Can mobile health technologies transform health care?. JAMA.

[2] Meskó B, Drobni Z, Bényei É, Gergely B, Győrffy Z (2017). Digital health is a cultural transformation of traditional healthcare. Mhealth.

[3] Mesko B (2023). The ChatGPT (generative artificial intelligence) revolution has made artificial intelligence approachable for medical professionals. J Med Internet Res.

[4] The effectiveness of World Bank support for community-based and -driven development. World Bank.

[5] Women’s & girls’ empowerment. The World Bank.

[6] Meskó B, Radó N, Győrffy Z (2019). Opinion leader empowered patients about the era of digital health: a qualitative study. BMJ Open.

[7] deBronkart D (2015). From patient centred to people powered: autonomy on the rise. BMJ.

[8] Mesko B (2020). Digital health technologies and well-being in the future. IT Prof.

[9] Poon EG, Jha AK, Christino M, Honour MM, Fernandopulle R, Middleton B, Newhouse J, Leape L, Bates DW, Blumenthal D, Kaushal R (2006). Assessing the level of healthcare information technology adoption in the United States: a snapshot. BMC Med Inform Decis Mak.

[10] Lewis DM (2020). Do-it-yourself artificial pancreas system and the OpenAPS movement. Endocrinol Metab Clin North Am.

[11] Melmer A, Züger T, Lewis DM, Leibrand S, Stettler C, Laimer M (2019). Glycaemic control in individuals with type 1 diabetes using an open source artificial pancreas system (OpenAPS). Diabetes Obes Metab.

[12] Lewis D, Leibrand S, #OpenAPS Community (2016). Real-world use of open source artificial pancreas systems. J Diabetes Sci Technol.

[13] Patients Included Charter Archive.

[14] Szasz TS, Hollender MH (1956). A contribution to the philosophy of medicine; the basic models of the doctor-patient relationship. AMA Arch Intern Med.

[15] Emanuel EJ, Emanuel LL (1992). Four models of the physician-patient relationship. JAMA.

[16] Coupe N, Mathieson A (2020). Patient and public involvement in doctoral research: impact, resources and recommendations. Health Expect.

[17] Foley L, Kiely B, Croke A, Larkin J, Smith SM, Clyne B, Pierce M, Murphy E (2021). A protocol for the evaluation of the process and impact of embedding formal and experiential public and patient involvement training in a structured PhD programme. J Multimorb Comorb.

[18] Riordan F, Racine E, Smith SM, Murphy A, Browne J, Kearney PM, Bradley C, James M, Murphy M, McHugh SM (2020). Feasibility of an implementation intervention to increase attendance at diabetic retinopathy screening: protocol for a cluster randomised pilot trial. Pilot Feasibility Stud.

[19] Frank L, Morton SC, Guise J, Jull J, Concannon TW, Tugwell P, Multi Stakeholder Engagement (MuSE) Consortium (2020). Engaging patients and other non-researchers in health research: defining research engagement. J Gen Intern Med.

[20] (2012). Briefing notes for researchers: public involvement in NHS, public health and social care research. NHS, Health and Social Care Research.

[21] Frank L, Basch E, Selby JV, Patient-Centered Outcomes Research Institute (2014). The PCORI perspective on patient-centered outcomes research. JAMA.

[22] (2022). Citizens and patients' advisory board. DZD.

[23] Skovlund PC, Nielsen BK, Thaysen HV, Schmidt H, Finset A, Hansen KA, Lomborg K (2020). The impact of patient involvement in research: a case study of the planning, conduct and dissemination of a clinical, controlled trial. Res Involv Engagem.

[24] Stepanian N, Larsen MH, Mendelsohn JB, Mariussen KL, Heggdal K (2023). Empowerment interventions designed for persons living with chronic disease—a systematic review and meta-analysis of the components and efficacy of format on patient-reported outcomes. BMC Health Serv Res.

[25] Aboumatar H, Pitts S, Sharma R, Das A, Smith BM, Day J, Holzhauer K, Yang S, Bass EB, Bennett WL (2022). Patient engagement strategies for adults with chronic conditions: an evidence map. Syst Rev.

[26] Samalin L, Honciuc M, Boyer L, de Chazeron I, Blanc O, Abbar M, Llorca PM (2018). Efficacy of shared decision-making on treatment adherence of patients with bipolar disorder: a cluster randomized trial (ShareD-BD). BMC Psychiatry.

[27] Barradell AC, Gerlis C, Houchen-Wolloff L, Bekker HL, Robertson N, Singh SJ (2023). Systematic review of shared decision-making interventions for people living with chronic respiratory diseases. BMJ Open.

[28] Hickmann E, Richter P, Schlieter H (2022). All together now—patient engagement, patient empowerment, and associated terms in personal healthcare. BMC Health Serv Res.

[29] Birkeland S, Bismark M, Barry MJ, Möller S (2022). Is greater patient involvement associated with higher satisfaction? Experimental evidence from a vignette survey. BMJ Qual Saf.

[30] Wensing M (2000). Evidence-based patient empowerment. Qual Health Care.

[31] Meskó B, deBronkart D (2022). Patient design: the importance of including patients in designing health care. J Med Internet Res.

[32] Reinius M, Mazzocato P, Riggare S, Bylund A, Jansson H, Øvretveit J, Savage C, Wannheden C, Hasson H (2022). Patient-driven innovations reported in peer-reviewed journals: a scoping review. BMJ Open.

[33] Dahlberg M, Lek M, Malmqvist Castillo M, Bylund A, Hasson H, Riggare S, Reinius M, Wannheden C (2023). Objectives and outcomes of patient-driven innovations published in peer-reviewed journals: a qualitative analysis of publications included in a scoping review. BMJ Open.

